# Oxidative stress and inflammation in diabetic nephropathy: role of polyphenols

**DOI:** 10.3389/fimmu.2023.1185317

**Published:** 2023-07-21

**Authors:** Qi Jin, Tongtong Liu, Yuan Qiao, Donghai Liu, Liping Yang, Huimin Mao, Fang Ma, Yuyang Wang, Liang Peng, Yongli Zhan

**Affiliations:** ^1^ Guang’anmen Hospital, China Academy of Chinese Medical Sciences, Beijing, China; ^2^ China-Japan Friendship Hospital, Institute of Clinical Medical Sciences, Beijing, China

**Keywords:** polyphenols, quercetin, resveratrol, curcumin, diabetic nephropathy, oxidative stress, inflammation

## Abstract

Diabetic nephropathy (DN) often leads to end-stage renal disease. Oxidative stress demonstrates a crucial act in the onset and progression of DN, which triggers various pathological processes while promoting the activation of inflammation and forming a vicious oxidative stress-inflammation cycle that induces podocyte injury, extracellular matrix accumulation, glomerulosclerosis, epithelial-mesenchymal transition, renal tubular atrophy, and proteinuria. Conventional treatments for DN have limited efficacy. Polyphenols, as antioxidants, are widely used in DN with multiple targets and fewer adverse effects. This review reveals the oxidative stress and oxidative stress-associated inflammation in DN that led to pathological damage to renal cells, including podocytes, endothelial cells, mesangial cells, and renal tubular epithelial cells. It demonstrates the potent antioxidant and anti-inflammatory properties by targeting Nrf2, SIRT1, HMGB1, NF-κB, and NLRP3 of polyphenols, including quercetin, resveratrol, curcumin, and phenolic acid. However, there remains a long way to a comprehensive understanding of molecular mechanisms and applications for the clinical therapy of polyphenols.

## 1 Introduction

Diabetes mellitus (DM) is a complicated chronic disease described by glucose dysregulation that is caused by absolute or relative defects, including type 1 diabetes (T1D) and type 2 diabetes (T2D). The global prevalence indicates that the number of DM is below half a billion individuals and is projected to increase by 25% and 51% in 2030 and 2045, respectively, putting enormous pressure on healthcare systems worldwide (1). As a complication of DM, diabetic nephropathy (DN), also called diabetic kidney disease (DKD), is the primary reason for chronic kidney disease (CKD) and even end-stage renal disease (ESRD), which is related to increased morbidity and mortality in patients with diabetes. Moreover, approximately 30%-40% of diabetic patients with DN (2). Patients with DN require maintenance dialysis or a kidney transplant. However, these therapy approaches bring a considerable economic and psychological burden and consume substantial medical resources. Therefore, promoting the amelioration of DN is of vital clinical significance (3).

The pathogenesis of DN is complicated and still needs to be determined. It has been proven that rigorous management of blood pressure and blood glucose cannot prevent the progression of DN to ESRD, nor can it prevent DN-related deaths. Developing the understanding and research of DN’s pathogenesis is crucial for expanding new methods for DN (4). Multiple pathways and mediators, including oxidative stress, inflammation, and angiotensin II (Ang-II), are involved in the incidence and progression of DN, of which oxidative stress is the most prominent (5, 6). Chronic hyperglycemia induces oxidative stress, promotes excess reactive oxygen species (ROS) production, reduces antioxidant capacity, induces the damage of oxidative stress in DNA and proteins, and stimulates the immune system to release inflammatory mediators and cytokines that affect glomerular capillaries and changes in renal tubular structure and function, thereby exacerbating renal and systemic damage. High glucose (HG)-induced overproduction of ROS is the primary initiator of cell damage in diabetes and its complications. For DN, although several therapies have been developed (7), such as Sodium-glucose cotransporter 2 (SGLT2), which effectively reduces inflammation and oxidative stress and has been shown to reduce the risk of major adverse events and progression of renal disease in patients with T2D, the ultimate treatment effect is not satisfactory (8).

Natural products, primarily from herbal sources, have long been explored as sources of drugs to treat a variety of major diseases. To date, significant efforts have been made to support and validate the potential effectiveness of natural and synthetic products in experimental studies and clinical applications, and indicating the antioxidant effects for kidney (9, 10). In preclinical studies, many natural products have recently been reported to alleviate kidney disease by modulating oxidative stress and inflammation (11). As a natural product, polyphenols are widely distributed in most plants and classified as phenolic acids, flavonoids, stilbenes, and lignans according to their structural properties (12).Nuclear factor E2-related factor 2 (Nrf2) is a significant regulator of antioxidant enzymes that protect the body from oxidative stress and inflammation, and Nrf2/antioxidant response element (ARE) signaling has been suggested as a promising target against oxidative stress-mediated diseases, such as diabetes and fibrosis. Dietary polyphenols, such as resveratrol, curcumin, and quercetin, can modulate Nrf2 signaling by mediating various kinases upstream of Nrf2 and also directly activate the expression of Nrf2 as well as downstream targets, such as heme oxygenase 1(HO-1) superoxide dismutase(SOD), and catalase (CAT), to inhibit oxidative stress and regulate inflammatory mediators (13–15).

Polyphenols exhibit anti-diabetic potential by reducing intestinal glucose absorption, increasing insulin secretion from pancreatic cells, and modulating gut microbiota and its metabolites (16–19). Natural polyphenols have wide-ranging pharmacological activities, particularly antioxidant activity and free radical-scavenging ability; moreover, they have been gradually used in the research of DN in recent years (20). This review provides a comprehensive summary of oxidative stress and oxidative stress-induced inflammation in DN and polyphenols targeting oxidative stress and inflammation to delay the progression of DN, which provides new insights into polyphenols as promising drug candidates for DN.

## 2 Oxidative stress and inflammation in DN

### 2.1 Role of oxidative stress

Oxidative stress is defined as an excessive accumulation of ROS caused by an imbalance between oxidants and antioxidants, resulting in oxidative damage to the body. Moderate ROS-mediated damage can usually be reversed, but excessive ROS production beyond the self-regulatory process often leads to irreversible damage to cellular function or death (21).ROS includes superoxide anion, hydrogen peroxide (H_2_O_2_), nitric oxide (NO), hydroxyl radical, and peroxynitrite (22). Mitochondria induce the production of ROS through the mitochondrial respiratory chain, which is the primary source of ROS. Other sources include nicotinamide adenine dinucleotide phosphate (NADPH) oxidase (NOX), changes in glucose metabolism, including polyol pathway flux, and changes in hemodynamics, advanced glycation end products (AGEs), and protein kinase C (PKC) (23, 24). Basal ROS levels are critical for maintaining various cellular tissue functions, such as gene expression, molecular transcription, and signaling transduction (25). However, excessive ROS accelerates pathological states, including the progression of DN, whereas the antioxidant defense system is activated to eliminate ROS generated from varied sources. Antioxidant enzymes include SOD, glutathione peroxidase (GSH-Px), CAT, glutathione reductase (GR), and paraoxonase (26). As markers of oxidative damage, malondialdehyde (MDA) and protein carbonyl can exacerbate oxidative damage in the body. Oxidative stress significantly affects the cause, onset, and process of DN, and hyperglycemia triggers the activation of the polyol pathway, AGEs, the receptor for advanced glycation end products (RAGE), and PKC. AGEs-RAGE signaling pathway activation potentiates the action of NOX and stimulates the production of ROS (27). Subsequently, ROS further interacted with NOX, aggravating the production of ROS (28). DN is associated with hypoxia, the generation of Ang II, and the production of ROS, leading to actin cytoskeleton reorganization of the podocyte, which induces podocyte injury, disrupts the glomerular filtration barrier and triggers proteinuria (29). Moreover, Ang II interferes with the formulation of ROS *via* the PKC/NADPH oxidase pathway, and ROS production is inhibited following the knockdown of PKC (30).

### 2.2 Oxidative stress and inflammation

Factors such as hyperglycemia and hypoxia induce oxidative stress and inflammatory responses. Additionally, along with inducing tissue oxidative stress damage, the release of ROS triggers the aggregation of inflammatory cells and the formulation of inflammatory cytokines, growth factors, and transcription factors related to the pathological process of DN (31). The infiltration of inflammatory cells, such as lymphocytes, neutrophils, and macrophages, contributes to kidney injury in DN (32). The recruitment and differentiation of immune-inflammatory cells are regulated by numerous inflammatory cytokines, such as nuclear factor-kappaB (NF-κB), NOD-like receptor family pyrin domain containing 3 (NLRP3), interleukin-1β (IL-1β), IL-6, tumor necrosis factor-α (TNF-α), monocyte chemoattractant protein-1 (MCP-1), intercellular adhesion molecule-1 (ICAM-1) and transforming growth factor-β (TGF-β). These factors are expressed in renal vascular endothelial cells (ECs), podocytes, mesangial cells (MCs), fibroblasts, monocytes, macrophages, and renal tubular epithelial cells (RTECs) (33). NF-κB, a key class of nuclear transcription factors, is involved in immune-inflammatory responses, oxidative stress, and apoptosis (34). NF-κB typically exists in the cytoplasm in an inactive form, such as a heterodimer. It is transported to the nucleus when stimulated by various factors, such as ROS. It binds to the NF-κB binding site to activate the transcription of NF-κB and promote the release of adhesion molecules and pro-inflammatory factors, including MCP-1, ICAM-1, TGF-β1, IL-1β, IL-6, and TNF-α, which can also regulate ROS level (35). HG induces the expression of receptor activator of NF-κB in podocytes, which increases the expression of NOX4 and P22phox and mediates the progression of DN (36).

NLRP3 inflammasome is a multimeric protein complex that induces physiological and pathological inflammatory responses by sensing pathogens, mediates cell pyroptosis, and is a critical downstream factor of NF-κB. Moreover, NLRP3 inflammasome can be triggered by ROS, aggravating the inflammatory cascade and cell damage. Additionally, it mediates the release of inflammatory factors, triggers mitochondrial dysfunction, and promotes ROS formation (37). The inhibition of NLRP3 inflammasome improves renal function by markedly inhibiting HG-induced activation of NF-κB p65, the production of mitochondrial ROS (38). In *in vivo* and *in vitro* studies, HG induces infiltration of inflammatory cells, including macrophages; stimulates the formulation of inflammatory factors, including IL-1β, IL-6, MCP-1, and TNF-α; triggers oxidative stress; induces MDA expression; inhibits Nrf2 pathway activation; and diminishes the production of downstream enzymes, such as HO-1, NADPH quinone oxidoreductase-1 (NQO-1), GSH-Px, SOD and CAT (39, 40).

High mobility group box 1(HMGB1), a typical intranuclear non-histone protein, reaches the nucleus by both active secretion and passive release. Once transported to the cytoplasm, it is involved in the immune response. In contrast, when released outside the cell, it can act as a potent inflammatory mediator, either alone or as part of a pro-inflammatory cascade response to stimulate the immune system (41). Interestingly, HMGB1 is regulated by ROS and NLRP3, translocates from the nucleus to the cytoplasm, binds toll-like receptor (TLR)4, activates NF-κB, and ultimately induces an inflammatory response (42).Relatively, HMGB1 induces mitochondrial dysfunction, such as increased ROS production and mitochondrial fission (43, 44).Conversely, H2O2 in the mitochondria and nucleus induces the formation and secretion of HMGB1, which promotes inflammatory responses (45).

Oxidative stress and inflammation interact in the pathological mechanism of DN, disrupting the structure and function of the kidney. The foot processes (FP) of podocytes are lost or fused, which leads to podocyte hypertrophy and reduced levels of podocyte-related proteins, such as nephrin and podocin (46) adhesion and platelet thrombosis on ECs, which triggers endothelial dysfunction (47) production of fibronectin (FN), and collagen IV; and accumulation of extracellular matrix (ECM) in MCs, which leads to glomerulosclerosis (48). The inhibition of NF-κB translocation improves mesangial cell fibrosis and subsequently improves ECM accumulation (49). Moreover, Epithelial-mesenchymal transition (EMT) is induced by TGF-β, which is closely related to tubulointerstitial fibrosis (50, 51). Many studies support that oxidative stress and inflammation are interdependent and interconnected processes that coexist in an inflammatory environment. Inflammatory cells release large amounts of ROS at the site of inflammation, leading to increased oxidative damage. In addition, many ROS and oxidative stress products enhance the pro-inflammatory response. Inhibition of inflammatory response and oxidative stress, reduction in the number of renal collagen fibers and glomerulosclerosis, restoration of glomerular barrier function, persistent reduction in proteinuria (52), and reduction in the generation of serum creatinine (Scr) and blood urea nitrogen (BUN), recover renal function and ameliorate the process of DN to ESRD (53, 54).

### 2.3 Renal impairment

Oxidative stress-induced kidney damage includes direct and indirect mechanisms. ROS may damage the DNA, proteins, and lipids of tissue cells, directly leading to pathological changes in the glomeruli, renal tubules, and renal interstitium, including cellular components, such as decreased levels of nephrin, podocin, and podocyte-related proteins (55), fusion, and disappearance of FP, hypertrophy, and apoptosis of podocyte (56). Oxidative stress exacerbates the release of oxidative stress markers and inflammatory mediators of vascular ECs, such as MDA, TNF-α, and IL-6 (57). These factors reduce NO and endothelial NO synthase expression levels, activating the endothelin-1 signaling pathway (58) and exacerbating vascular smooth muscle cell aging while inducing vascular calcification (59). ROS promotes the expression of collagen IV, FN, and laminin and the accumulation of ECM in HG-induced MCs (60, 61). Oxidative stress affects autophagy (62) and promotes lipid accumulation (63), EMT, and apoptosis in RTECs (64). Ultimately, oxidative stress exacerbates progressive glomerular damage, tubular atrophy, and interstitial fibrosis, leading to decreased renal function and renal failure (65). As the primary source of ROS, the mitochondria are also the organelles that supply energy to the body. The accumulation of ROS can damage the mitochondria, including an imbalance of mitochondrial fission and mitochondrial fusion, mitochondrial mitophagy, downregulation of respiratory chain complexes, insufficient ATP synthesis, interruption of mitochondrial membrane potential, the discharge of cytochrome-c and the generation of caspase-3, leading to mitochondrial damage (66), which are considered a significant factor in glomerular and tubular necrosis and apoptosis (67, 68).

**Figure 1:**
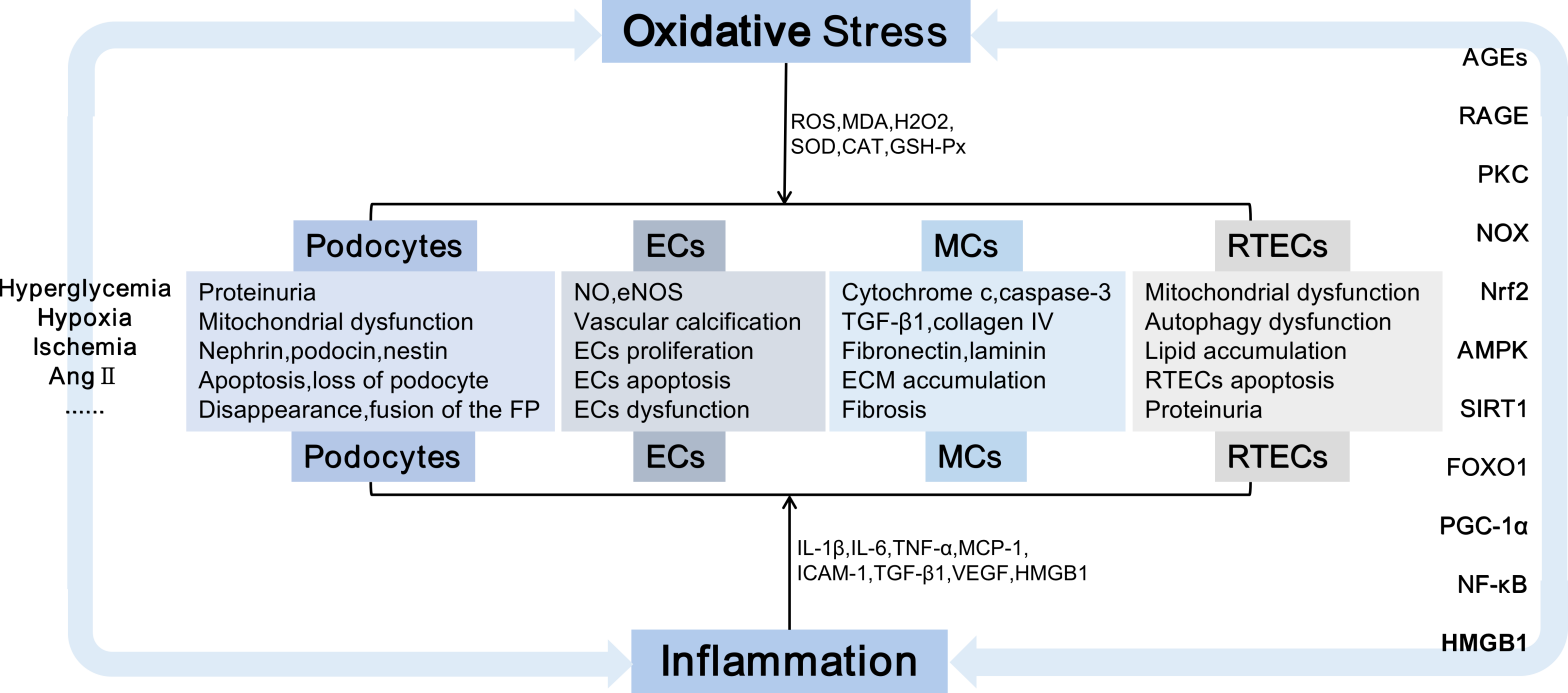
Kidney damage is caused by oxidative stress and inflammation. Damaged cells including podocytes, ECs, MCs, and RTECs, causing podocyte injury, the accumulation of extracellular matrix,epithelial-mesenchymal transition, cell apoptosis, etc., which eventually lead to irreversible glomerular fibrosis and tubular damage, exacerbate the progression of DN.Ang II, Angiotensin II; FP, Foot processes; ECs, endothelial cells; MCs, mesangial cells; RTECs, renal tubular epithelial cells; NO, nitric oxide; eNOS, endothelial nitric oxide synthase; TGF-β, transforming growth factor-β; ECM, extracellular matrix;IL-1, interleukin-1; TNF-α, tumor necrosis factor-α; MCP-1, monocyte chemoattractant protein-1; ICAM-1, intercellular adhesion molecule-1; VEGF,vascular endothelial growth factor; AGEs, advanced glycation end products; RAGE, receptor for advanced glycation end products; NOX, nicotinamide adenine dinucleotide phosphate oxidase; PKC, protein kinase C; Nrf2, nuclear factor E2-related factor 2;AMPK, AMP-activated protein kinase; SIRT1, Sirtuin-1; FOXO, forkhead transcription factor O; PGC-1α, peroxisome proliferator-activated receptor-γ coactivator-1α;NF-κB, nuclear factor-kappaB; DN, diabetic nephropathy;HMGB1,high mobility group box 1.

Indirectly, diversified signalling pathways, such as AGE-RAGE (69), Kelch-like ECH-associated protein(Keap1)-Nrf2 (70), AMP-activated protein kinase (AMPK)/Sirtuin-1 (Sirt1) (71), SIRT1-forkhead transcription factor O (FOXO) (72), Sirt1 (73), NF-κB (74) and HMGB1 could be induced by oxidative stress. These pathways not only lead to oxidative stress injury but also stimulate the release of inflammatory and apoptotic factors, which induce inflammation and apoptosis. Primarily, oxidative stress is linked with variations in renal hemodynamics and metabolism, and chronic hyperglycemia-induced oxidative stress can induce elevated levels of Ang-II and activation of PKC (75), glycolipid disorders (76) and insulin resistance (77), which are important stimulants that promote oxidative stress. Ang-II activates NOX, which produces superoxide, thereby promoting renal vascular remodeling and increasing preglomerular resistance (78).PKC promotes significant upregulation of NOX4 and enhances the generation of ROS (79). Additionally, PKC can lead to insulin resistance and exacerbate insulin dysfunction (80). Podocytes contain insulin receptors, maintain insulin signal transduction and podocyte function, specifically knock out insulin receptors on podocytes, decrease autophagy-related proteins, such as Beclin1 and light chain 3 (LC3), cause autophagy disorders and podocyte injury (81). HG can upregulate the expression of AGEs and RAGE; induce the activities of PKCα, PKCβ, and NOX4; promote the production of ROS; destroy the mitochondrial function of human renal MCs, and affect the renal function of db/db mice (79). ROS induces the accumulation of excess lipids and the formulation of growth factors, including TGF-β, vascular endothelial growth factor (VEGF), and inflammatory factors, which are essential factors that lead to renal dysfunction and subsequent nephropathy.ROS mediates HMGB1/mitochondrial DNA signaling, promotes TLRs activation and inflammatory responses, and exacerbates EMT in proximal renal tubular epithelial cells (82) (**Figure 1**).

## 3 Overview of polyphenols

### 3.1 Chemistry of polyphenols

**Figure 2:**
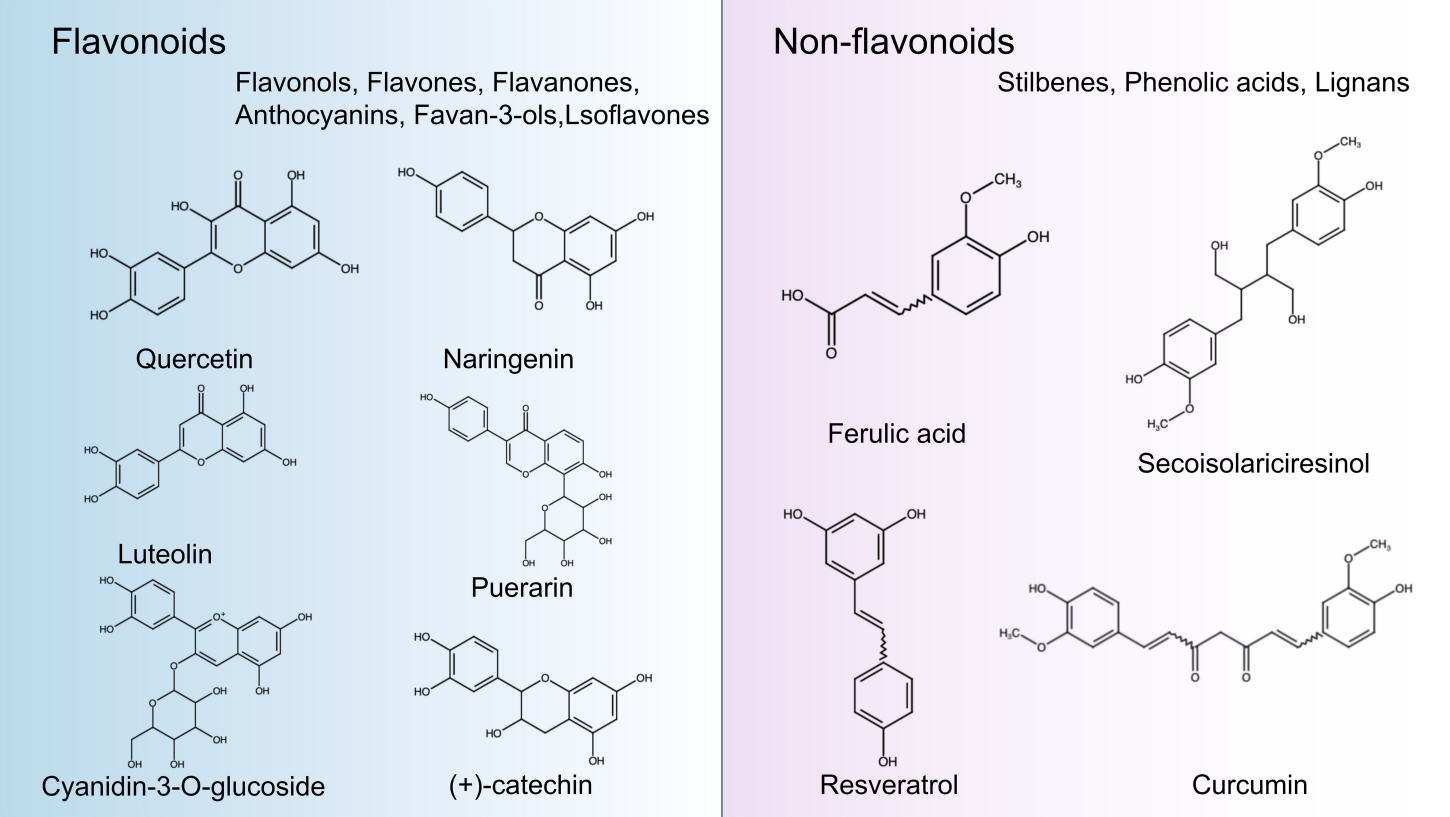
Classification and chemical structure of polyphenols. The structures of common polyphenol representatives (Created with BioRender.com.).

Polyphenols are secondary metabolites from various plants and are widely found in foods and beverages of plant origin, and more than 8000 polyphenols have been identified. Due to their antioxidant, anti-inflammatory and immunomodulatory activities, they play an essential role in the management of human health. In recent years, they have attracted much attention from nutritionists and food scientists due to their nutritional and therapeutic value (83). According to chemical structure, polyphenols can be divided into four major groups: Flavonoids, Stilbenes, Phenolic acids, and Lignans. Flavonoids comprise 15 carbon atoms, including two benzene rings (A Ring and B Ring) and a heterocycle (C Ring), abbreviated as C6-C3-C6, with the presence of flavan nucleus. Depending on the oxidation level of the C ring and hydroxylation patterns, flavonoids can be classified as flavonols, flavones, flavanones, anthocyanin, flavan-3-ols, and isoflavones (84). Non-flavonoids, including Stilbenes, Phenolic acids, and Lignans. Phenolic acids are the simplest compounds in the family of polyphenols because they have only one phenolic ring, such as ferulic acid, caffeic acid, and gallic acid (85). Stilbenes is a plant polyphenol with a diphenylethylene backbone. Resveratrol is the most representative compound among the stilbenes (86). Lignans are compounds produced by the oxidative dimerization of two phenyl propane units. Differences in the number of phenol units and how they are combined lead to different physical, chemical, and biological properties (87–89). In particular, the stability of polyphenols is also affected by many structural features, such as The hydrogenation of the C2=C3, hydroxylation, and methoxylation (90) (**Figure 2**).

### 3.2 The action of polyphenols with oxidative stress induced inflammation

**Figure 3:**
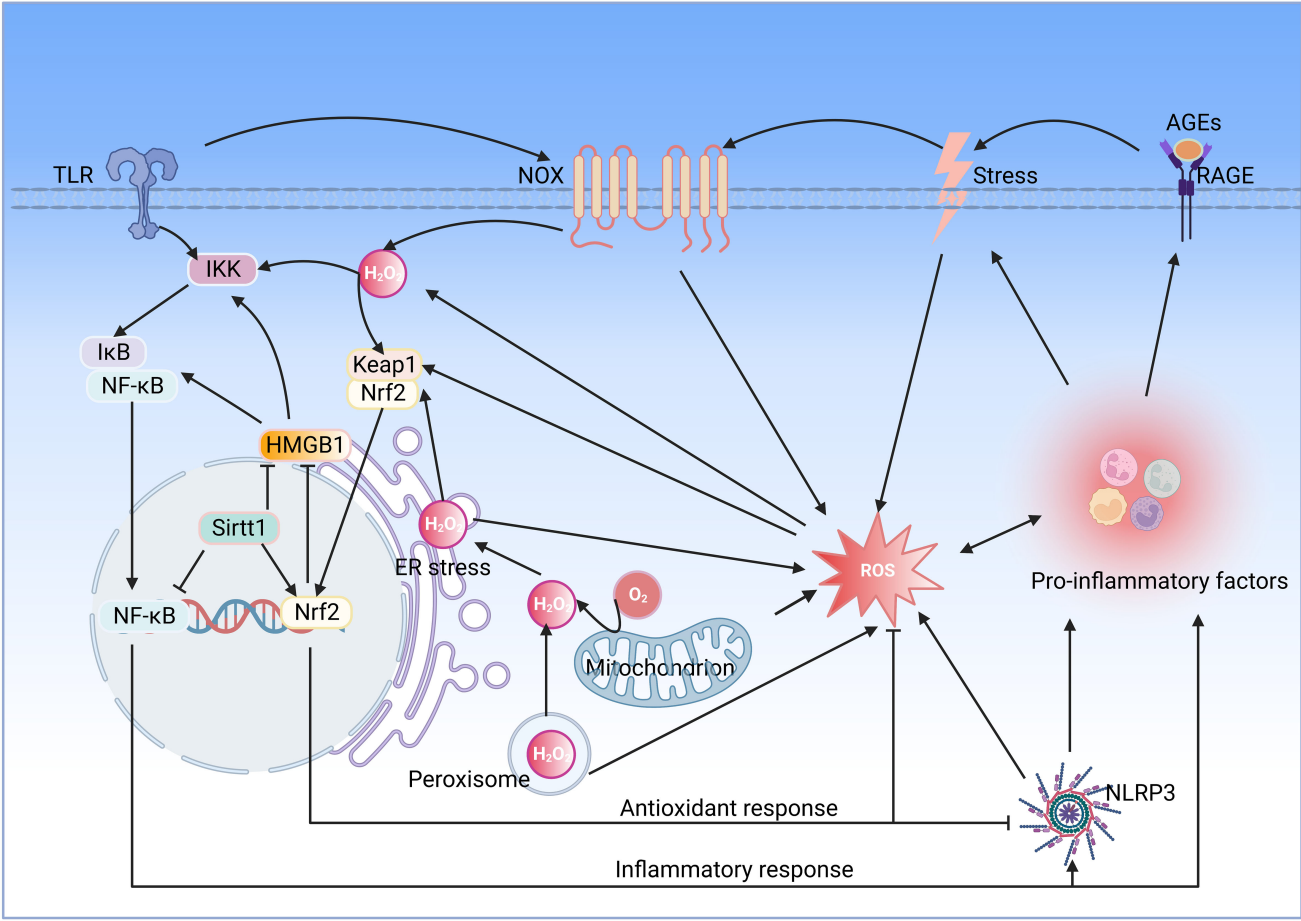
Interaction between oxidative stress and inflammation. Oxidative stress and inflammation interact with each other, and both of which constitute a vicious oxidative stress-inflammation cycle.TLR, toll-like receptor; IKK, IkB kinase; NF-κB, nuclear factor-kappaB; HMGB1, high mobility group box 1; Sirt1, sirtuin 1; Nrf2, nuclear factor E2-related factor 2; Keap, Kelch-like ECH-associated protein; NOX, NADPH oxidase; RAGE, receptor for advanced glycation end products; ER, endoplasmic reticulum; ROS, reactive oxygen species; NLRP3, NOD-like receptor family pyrin domain containing 3(Created with BioRender.com.).

Oxidative stress induced by ROS accumulation plays a pro-inflammatory role in various diseases, and NOX is considered a significant source of ROS. Inflammation inducers activate membrane recognition receptors of the innate immune system, such as TLR, which activate NOX and produce superoxide anion, which is rapidly converted to H_2_O_2_ by SOD, and H2O2 activates the Nrf2 signaling pathway to promote the release of antioxidant enzymes such as HO-1. More importantly, H2O2 activates inflammatory signaling pathways such as NF-κB, intensifying the release of pro-inflammatory mediators and stimulating inflammatory responses (91) (**Figure 3**).

Polyphenols have long been known for their antioxidant power to scavenge ROS. However, the impact of polyphenols goes far beyond this. Polyphenols can be indirectly anti-inflammatory through antioxidant effects or directly alleviate inflammation by modulating signaling pathways. The antioxidant capacity of polyphenols is mainly attributed to the direct scavenging of reactive oxygen species and the inhibition of reactive oxygen species production. Polyphenols inhibit the activity of NOX and significantly inhibit the production of superoxide anion (92). The phenolic ring structure of polyphenols can directly neutralize the free radicals generated by lipid peroxidation (93). Polyphenols can chelate metal ions, such as Fe3+, and prevent the conversion of H2O2 to highly toxic HO• (94). More importantly, polyphenols can promote the expression of antioxidant enzymes by enhancing endogenous antioxidant capacity induced by the Nrf2 pathway (95). Pomegranate polyphenols activate Nrf2 synapse, inhibit NF-κB suppression, reduce cell ROS production, and protect against drug-induced apoptosis (96). Resveratrol may activate the SIRT1/Nrf2 pathway to reduce inflammation and endoplasmic reticulum stress, effectively delaying the structural and functional decline of various tissues and organs due to aging (97). Polyphenols also enhance cellular antioxidant activity, inhibit pyroptosis and alleviate metabolic disorders and inflammatory responses through the Nrf2-NLRP3 axis (98).

**Figure 4:**
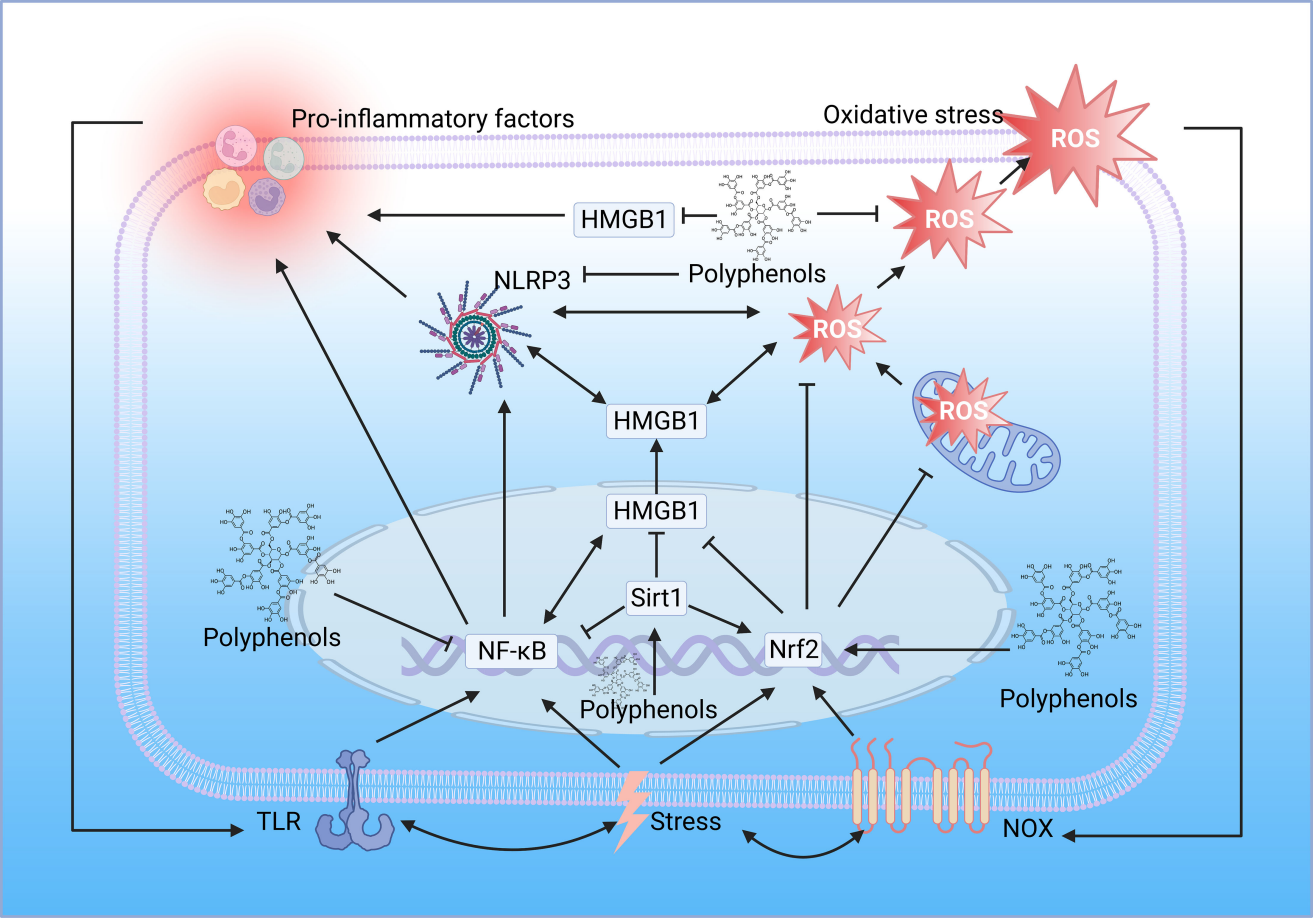
Polyphenols regulate oxidative stress and inflammation. The location of the polyphenol structure diagram in the figure is the critical node of polyphenol regulation. TLR, toll-like receptor; NF-κB, nuclear factor-kappaB; HMGB1, high mobility group box 1; Sirt1, sirtuin 1; Nrf2, nuclear factor E2-related factor 2; NOX, NADPH oxidase; ROS, reactive oxygen species; NLRP3, NOD-like receptor family pyrin domain containing 3 (Created with BioRender.com.).

While most studies suggest that polyphenols alleviate inflammation thanks to their antioxidant and free radical scavenging abilities, polyphenols can also directly modulate inflammatory signaling pathways. Many polyphenols, such as curcumin and resveratrol, have been reported to be potent inhibitors of NF-κB, inhibiting NF-κB translocation to the nucleus, inhibiting its binding from targeting DNA and subsequent transcription of pro-inflammatory cytokines, inhibiting phosphorylation or ubiquitination of signaling molecules, and inhibiting degradation of IκB (99). Polyphenols also target TLR4, the upstream of NF-κB, mitigating the inflammatory response through the TLR4/NF-κB signaling pathway (100). Resveratrol can suppress excessive inflammatory responses by interfering with HMGB1-mediated activation of the TLR4/NF-κB signaling pathway (101). Resveratrol suppresses HMGB1 expression through upregulation of miR-149, inhibits the ferroptosis formation pathway, and ameliorates injury (102). Also, resveratrol can mitigate downstream inflammatory responses by activating sirt1 and inhibiting the nucleoplasmic translocation and extracellular release of HMGB1 (103). Interestingly, a metabolomic study demonstrated the different roles of polyphenols in lipopolysaccharide-induced mouse embryonic fibroblast cells, with Theobroma cacao exerting mainly antioxidant properties and Lippia citriodora exerting anti-inflammatory effects mainly based on the reduction of pro-inflammatory cytokine and MCP-1 production, it was confirmed that the anti-inflammatory function of polyphenols could not be attributed to one mechanism of action but rather the coordination between different pathways and mechanisms (104) (**Figure 4**).

### 3.3 Polyphenols in DN

**Figure 5:**
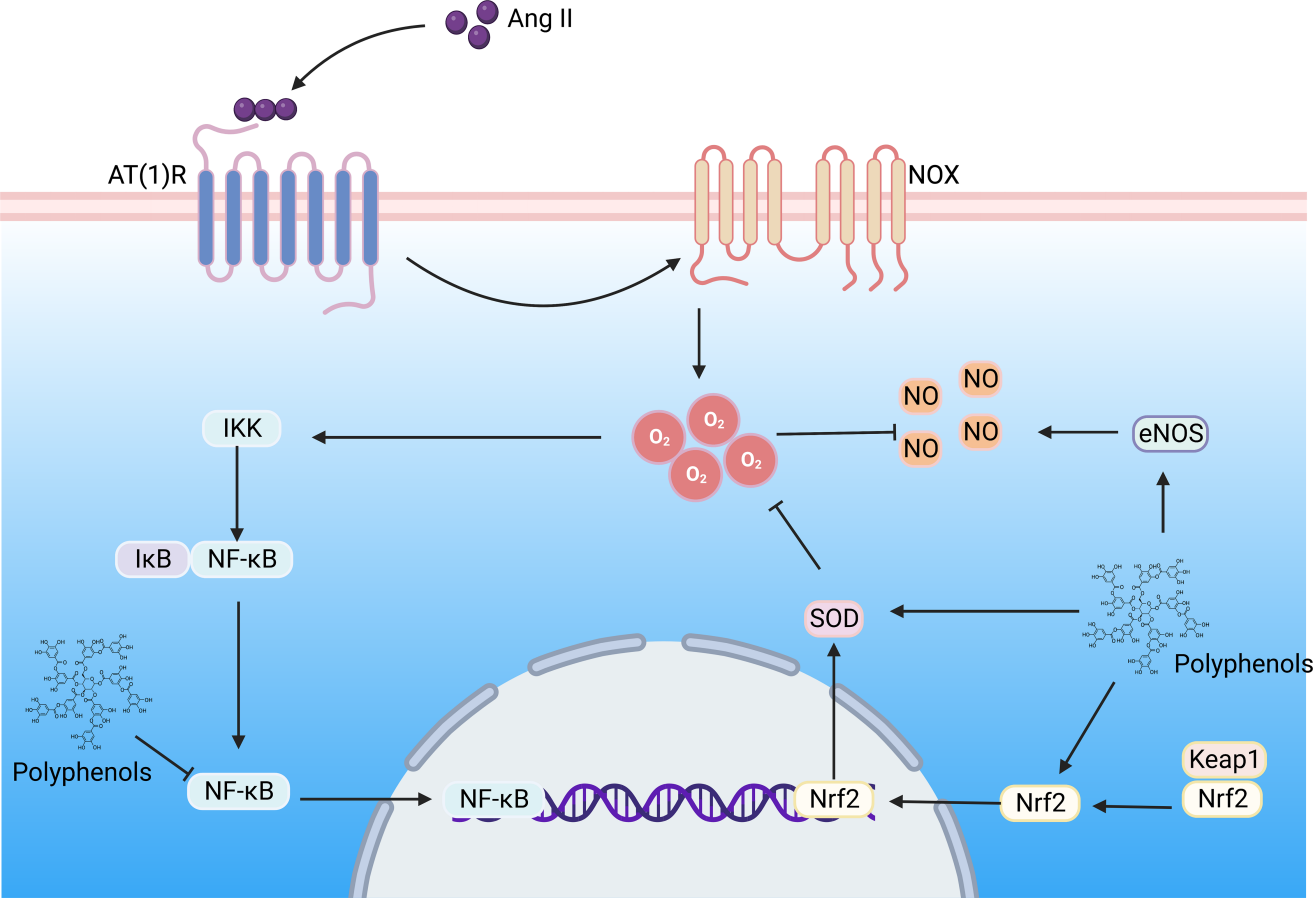
The polyphenols improve DN by regulating the Ang II signaling pathway. Hyperglycemia induces the formation of large amounts of Ang II, which binds to the AT(1)R) in the kidney, activating NOX, contributing to superoxide formation, triggering oxidative stress and inflammation, and resulting in renal vascular remodeling. Ang II, Angiotensin II; AT(1)R, Ang II type 1 receptor; NOX, NADPH oxidase; NF-κB, nuclear factor-kappaB; Nrf2, nuclear factor E2-related factor 2; NLRP3, NOD-like receptor family pyrin domain containing 3; IKK, IkB kinase; Keap, Kelch-like ECH-associated protein; SOD, superoxide dismutase; NO, nitric oxide;eNOS, endothelial nitric oxide synthase. (Created with BioRender.com.).

Some polyphenols have been widely used to repair molecules after free radical damage and modulate various dysregulated mediators and pathways, such as directly or indirectly blocking the production of ROS and alleviating the process of oxidative stress and inflammatory response, suggesting that polyphenols are an alternative approach to mitigate DN progression. In this review, “polyphenols”, “flavonoids”, “stilbenes”, “phenolic acids”, “lignans”, “flavonols”, “flavones”, “flavanones”, “anthocyanin”, “flavan-3-ols”, “isoflavones”, “diabetic nephropathy”, “diabetic kidney disease” as search topic words in Web of Science, and combining, de-duplicating to screen polyphenols as the primary intervention preclinical studies. (**Table 1**) (**Figure 5**).

**Table 1:** Effects of polyphenols on DN.

Name	Model	Dosage	Effect↓	Effect↑	Ref
Resveratrol	db/db	10mg/kg/d,12 w	FBG,SCr,BUN,TC,TG,MDA,desmin,	SOD,podocin,nephrin	(105)
	STZ	5mg/kg/d,8 w	FBG,SCr,TGF-β1,MDA,FN,NF-kB,	CAT,SOD, GPx,GSH,Nrf2,Sirt1,FoxO1	(106)
	db/db	20mg/kg/d,12w	TGF-β1, ECM, Bax	AMPK,PPARα,FoxO1,FoxO3a,PGC-1α,eNOS,Bcl-2	(107)
	STZ	5 mg/kg/d,30d	FBG,TNF-α, IL-1β, IL-6,NF-κB,superoxide anion,hydroxyl radical,NO,Keap1	CCR, SOD, CAT, GPx, Nrf2, GR, HO-1	(108)
	STZ	0.1,1mg/kg/d,7d	FBG,BUN,SCr,TG,the superoxide anion, carbonyl, IL-1β,	AMPK	(109)
	STZ	30mg/kg/d,16 w	FBG, MDA,UP-24H	CCR,SIRT1,SOD,CAT,FOXO3	(110)
	STZ	30mg/kg/day,12w	FBG,BUN,TC,proteinuria,MDA,	Mn-SOD,SIRT1,PGC-1α	(111)
	STZ	5,10 mg/kg/d,2w	FBG, SCr, BUN,	MDA, CCR, GSH, SOD, CAT,	(112)
	STZ	20 mg/kg/d,8w	FBG, urea,SCr, MDA,	SOD, GPX	(113)
	db/db	20 mg/kg/d,12w	FBG, SCr, albuminuria, type IV collagen, TGF-β1, Bax,caspase-3	CCR,AMPK, SIRT1,PGC-1α,FOXO3a,Bcl-2,SOD	(114)
	db/db	40 mg/kg/d,12w	FBG,SCr,BUN,MDA,NOX4,EMT,α-SMA,E-cadherin,TGF-β,IGF-1R	SOD, HRD1	(115)
Curcumin	STZ	80,130 mg/kg/d,60 d	FBG, SCr, urea, TC, TG, LDL-C, MDA, ROS,	HDL-C, TAC, NO	(116)
	OLETF	100 mg/kg/d,20w	FBG, UACR, MDA,	SOD,Nrf2/Keap1,HO-1,AMPK	(117)
	STZ	100 mg/kg/d,8w	FBG,Cr,BUN,UACR,PKC,NOX4,MDA,TGF-β1,CTGF, type IV collagen,FN,VEGF,p300	CCr, GPx	(118)
	STZ	15,30 mg/kg/d,2w	FBG,SCr,BUN,proteinuria,BUN,MDA	CCR, GSH, SOD, CAT	(119)
	STZ	10mg/kg/d,56d	24hUP,FN,TGF-β1,8-hydroxy-2’-deoxyguanosine		(120)
	STZ	50,100,200mg/kg/d, 8w	24hUP,UACR,MDA,ROS,caspase-3,Bax	CCR, SOD, Bcl-2	(121)
	STZ	100mg/kg/d,12w	FBG,urea,BUN,SCr,collagen II/III,TGF-β1,cytochrome-c,caspase-3,NF-κB,PKC,.NADPH oxidase	CCR, MnSOD, GSH, Bcl-2, Nrf2, FOXO-3a	(122)
Quercetin	STZ	50 mg/kg/d,8w	FBG,24hUP,BUN,Scr,TNF-α,IL-1β,AGEs,MDA	SOD, GSH-Px	(123)
	STZ	10mg/kg/d,8w	FBG,BUN,Scr,MDA,TG,LDL,ACR,ICAM-1	SOD, HDL	(124)
	STZ	50,100mg/kg/d,12w	BUN,TG,MDA,desmin,TGF-β1,Smad2, Smad3	Ccr, SOD, GSH, nephrin, podocin	(125)
	STZ	10 mg/kg/d,4w	FBG, TC, TG, proteinuria, urea,Cr, superoxide anions		(126)
	STZ	50mg/kg/d,5w	FBG,urea,BUN,SCr,TGF-β,TNF-α, IL-6	TAC, GSH and CAT	(127)
	STZ	100mg/kg/d,15d	FBG,urea,BUN,MDA,NF-kB,	SOD,CAT,SIRT1	(128)
Baicalin	STZ	40mg/kg/d,7d	TNF-α,NLRP3,MDA,P65,α-SMA,FN,TGF-β1	SOD,CAT,GPX	(129)
	db/db	400mg/kg/d,8w	ACR,AER,MDA, IL-1β, IL-6, MCP-1,TNFα	GSH-PX,SOD,CAT,Nrf2,HO-1,NQO-1	(39)
Naringenin	STZ	25,50mg/kg/d,4w	MDA	GSH,CAT,SOD	(130)
	STZ	5,10mg/kg/d,10w	TC,TG,LDL, VLDL, SCr,UA,TGF-β1,IL-1β	HDL,SOD,CAT,GSH	(131)
Silibinin	db/db	15,30 mg/kg/d,10w	BUN, SCr,UA, MDA	SOD, GSH-Px	(132)
Puerarin	STZ	20,40,80mg/kg/d,8w	FBG,BUN, Scr,24hUP,IL-6,TNF-α, ROS,NF-κB	MnSOD,CAT,SIRT1,FOXO1,PGC-1α	(133)
	STZ	20 mg/kg/d,8w	UACR,NOX4,NF-κB	SIRT1	(134)
	STZ	100mg/kg/d,7d	FP effacement,ROS,MMP-9,collagen IV	nephrin,podocin	(135)
Cyanidin-3-O-glucoside	STZ	10,20mg kg/d,8w	Scr,BUN,UA,ROS,MDA,TNF-α, MCP-1, IL-1β,IL-6,NF-κB, TGF-β1,Smad2,Smad3	SOD,GPX,CAT,Smad7	(136)
	db/db	10,20mg kg/d,12w	BUN,SCr,UA,ACR,collagen IV, FN,TGF-β1, MMP9,TG,TC,TNF-α,IL-1α, MCP-1	GCLC,GCLM,GSH	(137)
Apigenin	STZ	25,50mg/kg/d,30d	MDA,IL-1β,IL-6,TNF-α	Nrf2, HO-1,SOD,CAT	(138)
Luteolin	db/db	10mg/kg/d,12w	Fn, α-SMA, collagenI,collagen IV,IL-1β,IL-6,TNF-α,IL-17A,MDA,STAT3	SOD	(139)
	STZ	200 mg/kg/d,8w	BUN, SCr,24h UP,TC,TG,LDL,MDA	HDL,SOD,HO-1	(140)
Mangiferin	STZ	40mg/kg/d,30d	BUN,SCr,UA,ROS,Collagen,PKCs,MAPKs, NF-κB,TGF-β1,TNF-α,caspase 8,MMP	GSH,CAT, SOD, GPX,GR,Bcl-2, Bcl-xl	(141)
	STZ	15,30,60mg/kg/d,4w	FBG,TG,TC,BUN,SCr,UA,FN,collagen I,α-SMA,TNF-α,IL-6,IL-1β,MDA,ROS,TGF-β1,PI3K, Akt	SOD,CAT,GSH-Px	(142)
	STZ	15,30,60mg/kg,9w	AER,BUN,AGEs,RAGE,MDA	GSH	(143)
Ferulic Acid	STZ	50mg kg/d,8w	BUN,SCr,UACR,AGEs,ROS,NO,MDA,MAPK,TNF-α, IL-1β, and IL-6, MCP-1,ICAM-1,VCAM-1,NF-κB,	SOD2,CAT,beclin-1,LC3-II	(144)
	STZ	100mg kg/d,8w	BUN,Cr,FBG,TC,TG,MDA,NF-κB p65,TNF-α,TGF-β1,collagen IV	SOD, CAT, GPx, nephrin, podocin	(145)
	OLETF	10mg/kg/d,20w	24h UP, ACR, MDA, MCP-1, TGF-β1, collagen IV, ROS		(146)
Caffeic Acid	alloxan	50mg kg/d,7d	TC, TG, LDL,VLDL, MDA	HDL	(147)
Ellagic acid	STZ	50,100,150 mg/kg/d,4w	MDA,TNF-α,TLR4,IRAK4,TRAF6,IKKβ,NF- Kb	T-SOD	(148)
Chlorogenic acid	STZ	10, 20 mg/kg/d for 6 weeks	FBG, BUN, Cr, MDA	SOD, GSH-Px, CAT, Bcl-2	(149)
	STZ	10 mg/kg daily for 8 weeks,	BUN,proteinuria,MDA,IL-6,TNF-α,IL-1β,NF-ĸB	CCR, SOD, GSH-Px, Nrf2, HO-1	(150)

STZ, streptozotocin; FBG, fasting blood glucose; Scr, serum creatinine; BUN, blood urea nitrogen; TG, triglyceride; TC, total cholesterol; SOD, superoxide dismutase; MDA, malondialdehyde; TGF-β, transforming growth factor-β; GSH-Px, glutathione peroxidase; CAT, catalase; FN, fibronectin; NF-κB, nuclear factor-kappaB; Nrf2, nuclear factor E2-related factor 2; HO-1, hemoxygenase 1; Keap1, Kelch-like ECH-associated protein; SIRT1, Sirtuin-1; FOXO, forkhead transcription factor O; PPARα, peroxisome proliferator-activated receptor α; ECM, extracellular matrix; PGC-1α, peroxisome proliferator-activated receptor-γ coactivator-1α; NO, nitric oxide; eNOS, endothelial NO synthase; Bcl-2, B-cell lymphoma-2; Bax, BCL-2 associated X; CCr, creatinine clearance; IL-1β, interleukin-1β; TNF-α, tumor necrosis factor-α; GR, glutathione reductase; UP24H, 24h urine protein; EMT, epithelial-mesenchymal transition; α-SMA, α-smooth muscle actin; IGF-1R, IGF-1 receptor; HRD1, 3-hydroxy-3-methylglutaryl reductase degradation; HDL, high density lipoprotein; LDL, low density lipoprotein; TC, total cholesterol; VLDL, very-low-density lipoprotein; CCr, creatinine clearance; TOS, total oxidant status; TAC, total antioxidant capacity; UACR, urinary albumin to creatinine ratio; GGT, γ-glutamyltranspeptidase; CTGF, connective growth factor; ROS, eactive oxygen species; H2O2, hydrogen perxide; NADPH, nicotinamide adenine dinucleotide phosphate; NOX, NADPH oxidase; AGEs, advanced glycation end products; PKC, protein kinase C; AMPK, AMP-activated protein kinase; VEGF, vascular endothelial growth factor; 24hUP, 24-hour urinary protein; MnSOD, manganese superoxide dismutase; ACR, albumin/creatinine ratio; ICAM-1, intercellular adhesion molecule 1; OLETF, Otsuka-Long-Evans-Tokushima Fatty; AER, albumin excretion rate; NQO-1, quinone oxidoreductase-1; MCP-1, monocyte chemotactic protein-1; GCLC, glutamate-cysteine ligase catalytic subunit; GCLM,glutamate-cysteine ligase modifier subunit; MMP, mitochondrial membrane potential; TLR4, Toll-like receptor 4. Effect↓: decrease; Effect↑: increase.

#### 3.3.1 Flavonoids

##### 3.3.1.1 Quercetin

Quercetin, a polyphenol flavonoid, is ubiquitous in plants. Studies have shown that quercetin has a variety of biological activities, including scavenging free radicals and preventing lipid peroxidation in the body. Furthermore, it has anti-inflammatory properties and prevents diabetes complications (151, 152). Quercetin in streptozotocin (STZ)-induced DN rats can effectively reduce BUN and Scr levels, increase the formulation of nephrin and podocin while decreasing desmin in DN rats, relieve podocyte effacement (125) and improve renal pathological changes, including glomerular volume atrophy, high ECM and glycogen deposition, basement membrane thickening and tubulointerstitial fibrosis (123, 126). Further research illustrated that quercetin upregulated the formulation of SIRT1, activated the Nrf2/HO-1 pathway (128), inhibited the AGE-RAGE pathway (153), enhanced the expression of SOD, GSH-Px, and CAT (127), decreased the MDA, elevated the HDL (high density lipoprotein) content, decreased the expression of triglyceride (TG) and low-density lipoprotein (LDL), and regulated renal lipid accumulation. Additionally, quercetin decreased the expression of NF-κB, TNF-α, IL-1β, ICAM, and ICAM-1 (124). Vitro studies indicated that quercetin promoted the expression of SOD, GSH-Px, CAT, and SIRT1, and reduced the formulation of MDA, TNF-α, IL-1β, IL-6, TGF-β1, Smad 2/3, type IV collagen and laminin (154–156). There is consistent with a recent systematic study that reported that quercetin significantly decreased renal index, Scr, BUN, urine albumin, MDA, TNF-α, and IL-1β and increased the activities of SOD. Furthermore, CAT alleviated the degree of oxidative stress in animal models of DN (157). It is also proposed that the optimal dose for preclinical experiments is 90-150 mg/kg/day, and the administration time is 2-4 months (158).

##### 3.3.1.2 Puerarin

Puerarin is a natural isoflavone of Pueraria lobata (gegen), extensively found in food and Chinese herb medicine in East Asian countries. Puerarin has cardioprotective, antioxidant, and anti-inflammatory properties. Moreover, it exerts anti-diabetic activity by insulin secretion and maintaining metabolic homeostasis in STZ-induced diabetic mice (159). Furthermore, it can improve diabetes and other complications, including DN, by reducing the formation of AGEs and delaying oxidative stress (160). The SIRT1/FOXO1 signaling pathway is firmly related to the progression of DN. Studies have noted that activating the SIRT1/FOXO1 signaling pathway can increase SOD, CAT, and GSH-Px concentration and ameliorate renal injury in DN (161). Additionally, the SIRT1/FOXO1 signaling pathway can induce the expression of mitochondrial PGC-1α and improve mitochondrial function and ROS production (133). SIRT1 inhibited the NF-κB activity through deacetylation, and the expression of IL-6, TNF-α, and NOX4 was decreased, blood glucose, BUN, Scr, albuminuria, and urinary albumin to creatinine ratio (UACR) was ameliorated (134). Moreover, Puerarin directly diminished the formulation of ROS and FP effacement, restored the regular expression of nephrin and podocin of podocytes, decreased the formulation of matrix metalloproteinase-9 (MMP-9) and type IV collagen, and restored podocyte injury (135).

##### 3.3.1.3 Other flavonoids

In DN models, baicalin (162), apigenin, and silybin have been found to promote Nrf2 translocation into the nucleus. Genes induced by ARE are activated, such as HO-1 and NQO-1, accompanied by various antioxidant enzymes, such as GSH-Px, SOD, and CAT. However, the activity of MDA has been inhibited (39). Moreover, the concentration of IL-1β, IL-6, MCP-1, and TNF-α has been decreased, and the levels of FN and TGF-β1 that induced renal fibrosis have been reduced (129). Luteolin (163) promotes the expression of HO-1; reduces the expression of MDA, FN, α-SMA, collagen I, and collagen IV; and decreases the proliferation of MCs and excessive accumulation of ECM (140). The expression of STAT3 is highly correlated with oxidative stress and inflammatory response. Luteolin effectively inhibits the activation of STAT3 (139). Mangiferin inhibits the activation of the AGEs/RAGE axis, and PKCs reduce the phosphorylation of PI3K/Akt and improve the inflammation and oxidative stress in DN (141). Furthermore, similar to naringenin (131), the levels of TC, TG, LDL, and VLDL(very-low-density lipoprotein) were decreased, and the HDL level is raised, which improves lipid peroxidation by targeting oxidative stress and inflammation, cyanidin-3-O-glucoside (C3G) decreases the formulation of Smad2 and Smad3 while enhancing Smad7. It also improves renal pathological and functional changes, including the degree of renal fibrosis, SCr, BUN, urinary albumin, and ACR (137, 164). Eriodictyol can obstruct the production of NOX2, NOX4, directly shorten the production of ROS and MDA and protect MCs from HG stimulation by inhibiting the production of FN, collagen IV, and ECM (165). Notably, phloretin can directly restore nephrin and podocin levels and improve the disappearance of FP of podocytes (166). Furthermore, the inhibition of oxidative stress by apigenin *in vitro* has demonstrated the activation of Nrf2, HO-1, SOD, and CAT activities; downregulated the generation of TNF-α, IL-1β, and IL-6; inhibited the Nrf2, leading to the disruption of apigenin contrary to HG-induced oxidative damage was disrupted (167).

#### 3.3.2 Stilbenes

##### 3.3.2.1 Resveratrol

Resveratrol is a natural polyphenolic antioxidant. It has various biochemical and physiological properties, including antioxidant, anti-inflammatory, anti-diabetic, anti-obesity, cardiovascular protection, and anti-tumor features (168). Resveratrol exerts its renoprotective effects through multiple mechanisms, including reducing oxidative stress and AGE production, inhibiting endoplasmic reticulum stress and inflammation; improving lipotoxicity; stimulating autophagy (169); and activating a variety of pathways, including Nrf2, AMPK, SIRT1and FOXO3a. A systematic review showed that resveratrol could apply its antioxidant activity by reducing the production of MDA and restoring the action of SOD, CAT, and GSH-Px (170); decreasing 4-hydroxynonenal, an indicator of lipid peroxidation (171); and inhibiting the formation of ROS and lip peroxidation (172). Resveratrol activates antioxidant enzymes and decreases the secretion of superoxide anion, hydroxyl radical, TNF-α, IL-1β, IL-6, and NF-κBp65 by targeting the Nrf2-Keap1 signaling pathway; moreover, it improves the thickened basement membrane, leading to loss of FP (108). AMPK activation also has ameliorating effects on oxidative stress (169).AMPK inhibits HG-induced expression of NOX; decreases the content of FN and the proliferation of MCs; prevents EMT of RTECs (173, 174); restores podocyte-related proteins to normal, including nephrin, podocin, and desmin; and ameliorates mesangial matrix expansion, tubular basement membrane thickening, and glomerular hypertrophy in db/db mice of DN (105). Additionally, AMPK mediates the upregulation of SIRT1; activates the SIRT1/peroxisome proliferator-activated receptor α(PPARα)/FoxO pathway; heightens the generation of CAT, SOD, and GSH-Px; and decreases the expression of MDA, TGF-β1, FN, NF-kBp65, SCr, and urinary protein. In Silencing SIRT1, the protective effects are suppressed (106, 107, 110). Resveratrol elevates the concentration of Mn-SOD. Furthermore, it repairs the expression of SIRT1 and peroxisome proliferator-activated receptor-γ coactivator-1α(PGC-1α) and reestablishes the activity of respiratory chain complexes I and III and mitochondrial membrane potential. Furthermore, it inhibits the transport of Cyto C from the mitochondria to the cytoplasm and delays the progression of apoptosis in glomerular podocyte and tubular epithelial cells (111).

##### 3.3.2.2 Curcumin

Curcumin is an acidic polyphenol with unsaturated aliphatic and aromatic groups as the main chain, which is more common in turmeric (Curcuma longa L.) and Curcumae Rhizoma. The Pharmacological activities of Curcumin include antioxidant, anti-inflammatory, immunomodulatory, and anti-renal fibrosis. A previous study reported that Curcumin mediates the upregulation of GSH-Px, SOD, and other antioxidant factors by activating the antioxidant Nrf2/HO-1 pathway. Furthermore, it downregulates the expression of MDA and ROS effectively promotes improvement of the urinary protein excretion rate (175), elevates the expression of nephrin, and repairs podocyte injury (176). Additionally, Curcumin protects RTECs from HG-induced EMT *via* Nrf2-mediated upregulation of HO-1, which consequently knocks down Nrf2 and inhibits the upregulation of HO-1 (177). Curcumin activates the phosphorylation of AMPK, significantly increases the protein rate of Nrf2/Keap1, increases the activity of the antioxidant enzyme, and attenuates the expression of MDA, kidney injury molecule-1, and neutrophil gelatinase-associated lipocalin (116, 117). furthermore, it reduces the incidence of lipid accumulation, tubular dilatation, and glomerular sclerosis (178). Curcumin prohibited the activity of PKC and the expression of NF-κB, NADPH oxidase, collagen I/III, and TGF-β1. Also, it increases the protein levels of FOXO-3a, Nrf2, manganese SOD, GSH-Px, and B-cell lymphoma-2(Bcl-2); inhibits the generation of cytochrome-c and the production of caspase-3; and prevents damage to the mitochondria, renal tubules and mesangial cell (179). A systematic review of randomized displayed that Curcumin had some beneficial effects on various parameters, including inflammation or oxidative stress, in patients with renal disease (180). A randomized, double-anonymized trial assessing the redox status of dietary Curcumin supplementation (320 mg/d) in patients with non-diabetic or diabetic proteinuria confirmed that Curcumin enhanced the antioxidant capacity of diabetic proteinuric patients while attenuating lipid peroxidation in non-diabetic patients, suggesting a therapeutic effect of dietary Curcumin supplementation (181). *In vitro* studies have also found that Curcumin effectively reduces oxidized LDL isolated from human plasma-related markers such as conjugated diene, lipid peroxides, and lysolecithin, preventing oxidation and lipid modification of LDL (182).

#### 3.3.3 Phenolic acid

Multiple phenolic acids have been used, including ferulic, chlorogenic, caffeic, ellagic, etc. Phenolic acids have various functions, such as regulating blood sugar and lipids, anti-oxidation, anti-inflammatory, and anti-fibrosis. Hyperglycemia triggers oxidative stress, leading to the formulation of mitogen-activated protein kinases (MAPK). Ferulic acid inhibits the activation of MAPKs and decreases the expression of ROS, NO, carbonyl, MDA, and inflammatory factors and adhesion molecules, including NF-κB, TNF-α, IL-1β, and IL-6, MCP-1, ICAM-1 and vascular cell adhesion molecule-1. Moreover, it increases the levels of Beclin-1 and LC3-II (144). Long-term treatment with ferulic acid significantly downregulated the expression of p-NF-κB p65, TNF-α, TGF-β1 and type IV collagen protein, and MDA in renal tissue; increased the activity of SOD, CAT, GPx; and upregulated the levels of nephrin and podocin, which improved podocyte injury and reduced the serum levels of BUN, Cr, fasting blood glucose (FBG), TC (total cholesterol), TG by alleviating oxidative stress, inflammation and fibrosis in STZ-induced DN rats (145). Chlorogenic acid and caffeic acid increased renal formulation of Nrf2 and HO-1 and increased the activity of SOD and GSH-Px. Furthermore, they decreased the formulation of NF-ĸB, IL-6, TNF-α, and IL-1β (150, 183). Chlorogenic acid can also alleviate endoplasmic reticulum stress, promote the formulation of anti-apoptosis factors, such as Bcl-2, attenuate the proliferation of MCs and mesangial expansion, and reduce the levels of FBG, BUN, and Cr (149). Caffeic acid reduces the activation of NOX; decreases the generation of ROS; and heightens the action of antioxidant enzymes, including T-SOD, GPx, and GSH (184), thereby reducing the levels of MDA (148). Mainly, ellagic acid significantly inhibits the activation of renal NF-κB, IL-1β, IL-6, and TNF-α. Additionally, it decreases the generation of TGF-β and fibronectin in renal tissue (185).

## 4 Conclusion and prospect

This review summarizes oxidative stress and oxidative stress-associated inflammation that lead to changes in renal cell structure and function in DN. Oxidative stress is recognized as a powerful mechanism for the occurrence and progression of DN.DN, a common complication of DM, has become a significant cause of end-stage renal disease. Even though the pathogenesis of DKD is complex, oxidative stress has been proposed to be central to the pathogenesis of DKD. Hyperglycemia activates signaling pathways such as AGE/RAGE and Ang II, which induce large production of ROS. Large amounts of ROS activate NF-κB, TGF-β1, AMPK, and other pathways, which induce renal inflammation, autophagy, and fibrosis, triggering abnormal kidney structure and function. In addition, oxidative stress also interacts with other factors in disease progression. ROS are not only messengers of various signaling pathways but also regulators of various cellular metabolism, proliferation, differentiation, and apoptosis. Oxidative stress is accompanied by tissue inflammation, apoptosis, and tissue fibrosis, and in particular, oxidative stress and inflammation are considered to be in a causal relationship, with many factors exacerbating the progression of DKD (186). Oxidative stress induces excessive production of ROS, activates signaling pathways and transcription factors, mediates the infiltration and recruitment of inflammatory cells, such as macrophages and monocytes, and promotes the production of inflammatory factors. Conversely, the formulation of inflammatory mediators can further produce ROS, which aggravates oxidative stress, and oxidative stress and inflammation collaborate to affect the progression of DN. As a natural compound with antioxidant and anti-inflammatory properties, polyphenols can ameliorate or reverse cell damage and pathological changes of DN and are considered to be attractive drugs against DN by targeting signaling pathways, including Nrf2, NF-κB, AMPK, and SIRT1. However, the complex regulatory mechanism of polyphenols in DN has not been thoroughly demonstrated, and inhibition of renal damage and restoration of structure and function should be the core of alternative polyphenol therapy. Therefore, more studies are needed to elucidate polyphenols and how to repair kidney injury and improve kidney function.

Polyphenols exhibit various biological activities, such as antioxidant and anti-inflammatory. Multiple clear experimental evidence suggests that long-term intake of polyphenols can effectively improve the progression of chronic diseases, including DN. However, there are still some limitations, polyphenols are famous for their potent antioxidant properties, but in some cases, such as in reaction systems rich in redox-active metals (e.g., iron, copper), flavonoids have a pro-oxidant effect, reducing the clearance of ROS and inducing mitochondrial toxicity (187). In addition, the lack of pharmacokinetic measurements of ingested polyphenols and the correct dose remains a challenge, so the application should be tailored to the purpose and the selection of the appropriate dose and mode of action. Moreover, the evaluation of the efficacy of polyphenols in combination with other conventional treatments for DN and the specific mechanism of action remains to be investigated. Many natural products did not enter clinical trials or were terminated due to reduced oral utilization or activity, which may limit their widespread clinical application, and further studies are needed to overcome these limitations and to bridge the gap between preclinical and clinical studies of natural products. It is worth noting that the clinical translation of DN therapies from natural products is still a long process.

## Author contributions

QJ: Conceptualization, Writing-Original draft, Investigation, Visualization. TL: Conceptualization, Investigation. YQ: Investigation. DL: Investigation. LY: Investigation. HM: Investigation. FM: Investigation. YW: Investigation. LP: Writing-review & editing. YZ: Writing-review & editing.

## Glossary

**Table:**

DM	Diabetes mellitus
T1D	type 1 diabetes
T2D	type 2 diabetes
DN	Diabetic nephropathy
DKD	diabetic kidney disease
CKD	chronic kidney disease
ESRD	end-stage renal disease
Ang II	Angiotensin II
ROS	reactive oxygen species
HG	high glucose
SGLT2	sodium-glucose cotransporter 2
Nrf2	nuclear factor E2-related factor 2
ARE	antioxidant response element
HO-1	heme oxygenase 1
SOD	superoxide dismutase
CAT	catalase
H_2_O_2_	hydrogen perxide
NO	nitric oxide
NADPH	nicotinamide adenine dinucleotide phosphate
NOX	NADPH oxidase
AGEs	advanced glycation end products
PKC	protein kinase C
GSH-Px	glutathione peroxidase
GR	glutathione reductase
MDA	malondialdehyde
RAGE	receptor for advanced glycation end products
NF-κB	nuclear factor-kappaB
NLRP3	NOD-like receptor family pyrin domain containing 3
IL-1β	interleukin-1β
TNF-α	tumor necrosis factor-α
MCP-1	monocyte chemoattractant protein-1
ICAM-1	intercellular adhesion molecule-1
TGF-β	transforming growth factor-β
ECs	endothelial cells
MCs	mesangial cells
RTECs	renal tubular epithelial cells
NQO-1	NADPH quinone oxidoreductase-1
HMGB1	high mobility group box 1
TLR4,	Toll-like receptor 4
FP	foot processes
FN	fibronectin
ECM	extracellular matrix
EMT	epithelial-mesenchymal transition
BUN	blood urea nitrogen
Keap1	Kelch-like ECH-associated protein
AMPK	AMP-activated protein kinase
Sirt1	Sirtuin-1
FOXO	forkhead transcription factor O
STZ	streptozotocin
HDL	high density lipoprotein
TG	triglyceride
LDL	low density lipoprotein
UACR	urinary albumin to creatinine ratio
TC	total cholesterol
VLDL	very-low-density lipoprotein
UACR	urinary albumin to creatinine ratio
MMP-9	matrix metalloproteinase-9
C3G	Cyanidin-3-O-glucoside
ACR	albumin/creatinine ratio
PPARα	peroxisome proliferator-activated receptor α
PGC-1α	peroxisome proliferator-activated receptor-γ
Bcl-2	B-cell lymphoma-2
MAPKs	mitogen-activated protein kinases
FBG	fasting blood glucose.
